# Attention-Based Context Aware Network for Semantic Comprehension of Aerial Scenery

**DOI:** 10.3390/s21061983

**Published:** 2021-03-11

**Authors:** Weipeng Shi, Wenhu Qin, Zhonghua Yun, Peng Ping, Kaiyang Wu, Yuke Qu

**Affiliations:** School of Instrument Science and Engineering, Southeast University, Nanjing 210096, China; bowenroom@seu.edu.cn (W.S.); zhonghuayun@seu.edu.cn (Z.Y.); pingpeng@seu.edu.cn (P.P.); wukaiyang@seu.edu.cn (K.W.); 220203549@seu.edu.cn (Y.Q.)

**Keywords:** pattern recognition, computer vision, semantic segmentation, self-attention, deep learning, convolutional neural network, remote sensing

## Abstract

It is essential for researchers to have a proper interpretation of remote sensing images (RSIs) and precise semantic labeling of their component parts. Although FCN (Fully Convolutional Networks)-like deep convolutional network architectures have been widely applied in the perception of autonomous cars, there are still two challenges in the semantic segmentation of RSIs. The first is to identify details in high-resolution images with complex scenes and to solve the class-mismatch issues; the second is to capture the edge of objects finely without being confused by the surroundings. HRNET has the characteristics of maintaining high-resolution representation by fusing feature information with parallel multi-resolution convolution branches. We adopt HRNET as a backbone and propose to incorporate the Class-Oriented Region Attention Module (CRAM) and Class-Oriented Context Fusion Module (CCFM) to analyze the relationships between classes and patch regions and between classes and local or global pixels, respectively. Thus, the perception capability of the model for the detailed part in the aerial image can be enhanced. We leverage these modules to develop an end-to-end semantic segmentation model for aerial images and validate it on the ISPRS Potsdam and Vaihingen datasets. The experimental results show that our model improves the baseline accuracy and outperforms some commonly used CNN architectures.

## 1. Introduction

In the domain of remote sensing, a key aspect is for researchers to understand images correctly. The target is to design a model to recognize the detailed regions in the RSI (remote sensing images). The classification of each pixel in an image, also known as semantic segmentation in the field of computer vision, can distinguish each tiny target object in an aerial image. Using the approach of semantic segmentation to better grasp the semantic information in images can assist researchers in making breakthroughs in the following areas: keeping track of changes in buildings [1,2,3], extracting information about road networks [4,5,6], urban planning [7,8], zoning of urban land parcels [9,10,11], water coverage surveys [12,13], and so on. With the progressive and dramatic improvement of computing power over the years, deep learning-based methods are playing an essential role in addressing the issues of remote sensing. Different from the traditional methods that apply the hand-crafted points to distill information, FCN-based semantic segmentation algorithms, which can recognize each pixel in an image end-to-end and can efficiently acquire the feature information, have made significant breakthroughs over the years and are well implemented in the field of autonomous driving and virtual simulation. The FCN-based CNN architecture can be roughly divided into four types [14]: image pyramid, encoder-decoder, context module, and spatial pyramid pooling.

**Figure 1:**
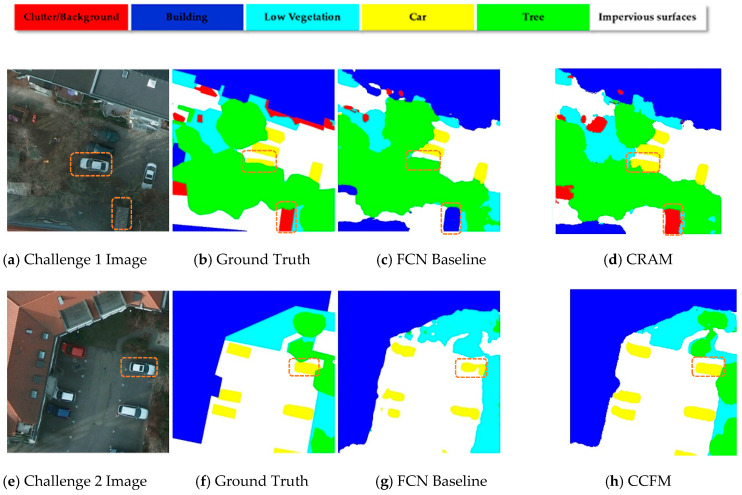
Illustration of challenges in the task of semantic segmentation for aerial images.

We consider that there still remain two major challenges to semantic segmentation on aerial images as Figure 1 illustrates. Firstly, there exists a complex relationship between various classes in the RSIs. Distinct from the Cityscapes [15] dataset that has usually been standardized as a benchmark test for semantic segmentation algorithms, aerial images are often characterized by high resolution and rich semantic information per pixel (for example, the Potsdam dataset [16] released by ISPRS has a single frame resolution of 6000 × 6000 and a GSD of 5 cm), which leads to the sophisticated correlation between different output categories in aerial images. Although the FCN-like CNN architecture can obtain intensive semantic information from the input, it is difficult to cope with the complicated scenes owing to the limited and localized receptive field. In addition, objects of different sizes in diverse regions of an image do not identically contribute to the final output. Consequently, the issue of mismatching relationships between classes often emerges in the output result.

Secondly, it is challenging to accurately distinguish the boundary of specific objects in aerial images from their surroundings. Take the Potsdam dataset as an example, the labels of low vegetation and trees, cars, and cluttered backgrounds, etc., are often confused by computer vision models because they have similar features from a local perspective. The FCN-like CNN architecture gradually loses spatial information in the decoding process, and this information is not well recovered in the upsampling process. This could lead to problems with the modeling algorithm in accurately classifying the objects in the photo and finely segmenting the outer edge.

In order to tackle both challenges, we developed and applied CRAM and CCFM based on HRNET, which links convolutional blocks of different resolutions in parallel and enables them to communicate with each other, so that the network with the fusing information can retain robust, high-resolution representations during the feature extraction process. Therefore, we have selected HRNET as our backbone here to facilitate the steady flow of complex information across multiple branches of the network. CRAM first selects $N_{C}$ small regions from the image by means of 1 × 1 convolution ($N_{C}$ is the number of recognized species) and roughly delineates the location of pixels belonging to the same category, after which the data are fused and interacted by the operation of the pooling layer, which enables the model to generate multi-scale semantic representations. As shown above, in the image of Challenge 1, CRAM can accurately handle the relationship between output category and location due to the feature representations extracted from subregions and can conduct proper semantic segmentation for hidden vehicles under trees that are not recognized in the baseline. CCFM utilizes a nonlocal strategy to accurately segment the edge of each object by capturing the contextual semantic information from long-range dependencies and local area pixels, while saving GPU memory. As shown in the challenge 2 image above, the semantically segmented vehicle is more complete, and the edge is sharp.

In summary, our contributions are as follows:

- Based on the backbone of HRNET, we integrate CRAM to investigate the relationship between semantic segmentation output classes and patch regions in aerial images, which is useful for solving mismatch issues.
- We introduce CCFM that leverages an attentional mechanism to better interpret the relationship between classes and specific pixels, facilitating the acquisition of semantic information from long dependencies and providing a multi-scale contextual representation for the semantic segmentation task in aerial images, thus allowing for a detailed identification of the outlines.
- Based on HRNET, CRAM, and CCFM, we propose an end-to-end context aware network for semantic comprehension of aerial scenery. We have also conducted a series of ablation studies and compared these with the state-of-the-art algorithms in the industry to demonstrate the effectiveness of our model.

## 2. Related Work

### 2.1. Semantic Segmentation

Semantic segmentation in deep learning is an important task in visual understanding. A fully convolutional neural network [17] has made great progress in the field of semantic segmentation by making use of learning and the representation ability of classifiers. In order to identify objects at different scales, there are four mainstream methods: image pyramid structure, encoder - decoder structure, semantic module method, and spatial pyramid pooling method. UNet [18], UNet++ [19], DeepLabV3+ [20], and DFN [21] utilize the structure of encoder and decoder, in which the encoder structure is used to acquire deep semantic information in the image, and the decoder structure can gradually recover the spatial information lost in the process of downsampling. DeepLabV3 [14] adopts the astrous spatial pyramid pooling method, which can capture convolution information at different scales. Through the scene parsing network and combined with the pyramid pooling module, PSPNet [22] can obtain comprehensive semantic information between different regions of the image. The GCN [23] network proposes a refinement method based on large kernels and residual networks to further refine the boundary structure of objects. DUC-HDC [24] can effectively enlarge the receptive field and decrease the gridding effect. Through the spatial path and context path, BiseNet [25] can efficiently obtain the largest receptive field as possible and retain the feature information of the image at the same time. DenseASPP [26] can acquire different features at multi-scales without more parameters through joining astrous convolutional layers densely. HRNet [27] maintains a high resolution in the process of feature extraction. By connecting convolution operations from high to low in parallel and exchanging information between different operations, the model can maintain better semantic information and more precise spatial information.

### 2.2. Attention Model

The attention model was first widely used in neural machine translation [28]. Later, it made better breakthroughs in many fields and has become an important part of neural network structures. Attention U-Net [29] introduces the attention gate mechanism model to medical images, so that the neural network structure model can focus on objects with irregular shapes and sizes in the target image. In this way, semantic segmentation tasks can refine the edges of inconspicuous classes. Non-local neural networks [30] capture long-range dependence by calculating the interaction between two-pixel locations. The author also pointed out that self-attention can be regarded as one of the non-local methods; therefore, self-attention is not only applied to neural machine translation, but can also be applied to the generalization of visual information processing. SAGAN [31] applies the self-attention model in the field of image generation, capturing more information from all feature map positions, and the discriminator can establish the correlation between different pixels even if under long-distance conditions, which is an improvement of the effect in model generation and discrimination. PSANet [32] designs a point-wise spatial attention network, which uses adaptive attention masks to connect each position on the feature map, thereby lowering local neighborhood constraints. DANet [33] includes a space and channel attention module on the basis of FCN. The space attention module can aggregate different features at different positions, and the channel attention module can integrate the correlation features between different channels to yield more precise results. OCNet [34] employs the strategy of aggregating contextual information using ground truth values to supervise the learning of target areas, uses the corresponding object context representation to characterize the pixels, and then calculates the relationship between each pixel and each target area via object-contextual representation to extend the representation of each pixel. CCNet [35] proposed a new crisscross attention model to obtain the context information of nearby pixels. By means of the recurrent cross-attention module, CCNet can obtain the dependency relationship between all pixels, while ensuring the calculation accuracy, and greatly reduces the occupation of GPU memory. ECANET [36] put forward a local cross-channel interaction method without reducing the dimension, which can achieve fast and efficient convolution operations. Ref. [37] put forward a task-wise attention module to acquire task-specific feature representation for few-shot image classification. Ref. [38] designed the Expectation-Maximization Attention (EMA) module which is robust to different input data along with a reduction in the memory cost. Supervised by a discriminative objective function, [39] brings forward D-CNNs that can improve the performance of scene classification in remote sensing.

### 2.3. Semantic Segmentation of Aerial Imagery

For semantic segmentation of aerial images, multi-scale object recognition and fast location of objects in images are the two vital issues. Because of the small size of objects in aerial images, the utilization of deep learning techniques can greatly improve the accuracy and efficiency of aerial image recognition. Ref. [40] suggests applying a multi-channel attention approach integrated with multi-layer features and residual convolution module for efficient object recognition and location. TreeUNet [41] uses Tree-CNN block coupled with a concatenating operation to fuse and interchange the information of the feature map so that different parameter information can be shared in the multi-layer neural network. Ref. [42] uses HRNET as the backbone to acquire high-resolution global features without going through the decoding layer and combines the adaptive spatial pooling (ASP) module to collect and fuse the local information. Based on the attention mechanism, HMANet [43] makes use of the extended class augmented attention module (CAA), class channel attention module (CCA), and region shuffle attention module (RSA) to boost the ability of the network to locate and classify very small regions. DE-Net [3] combines the downsampling module, encoding module, compression module, and upsampling module in the similar form of an Inception network, so that DE-Net can accurately detect and extract the outer edge of the building in the aerial image. Ref. [44] proposed transfer learning and a recurrent fully convolution network on the basis of multiple 3D filters to process the change information in the image. Ref. [45] advocated a new method of a multi-scale super-pixel guidance filter based on feature selection. By processing high-dimensional and multi-scale guidance filters, considering the boundary and internal consistency of objects, it can better explain the geometric information of land covering objects in high-definition images. Ref. [2] exploited the inferred attention weight of the reweighted FCN, along the spatial and channel dimensions under the attention mechanism to integrate the low-level feature map into the high-level feature map in a goal-oriented way. Ref. [46] proposes spatial and channel relation modules to have an analysis on the global relationship between different feature maps.

## 3. Methods

### 3.1. Overview

**Figure 2:**
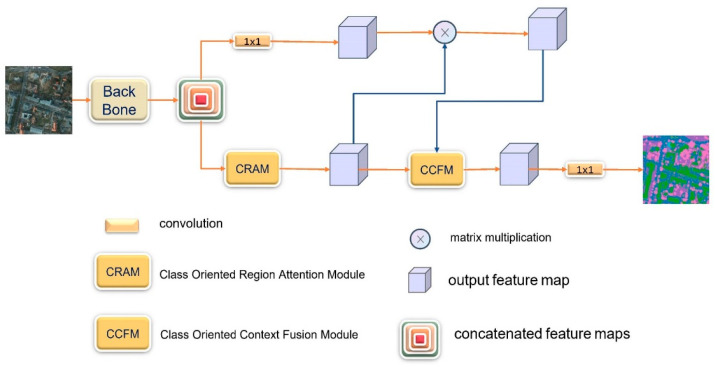
The pipeline of our method.

Figure 2 shows our end-to-end network model for remote sensing semantic segmentation. The backbone network is HRNET, which uses CRAM to explore the relationship between the output classes of semantic segmentation and the patch regions and the recurrent CCFMs to explore the relationship between the output classes and the specific pixels in the image. CRAM is applied to resolve the mismatch problems in the process of semantic segmentation of high-resolution aerial images. The model can collect information from different branches and has a global understanding of the scene using CCFM.

### 3.2. Backbone: High-Resolution Network (HRNet)

**Figure 3:**
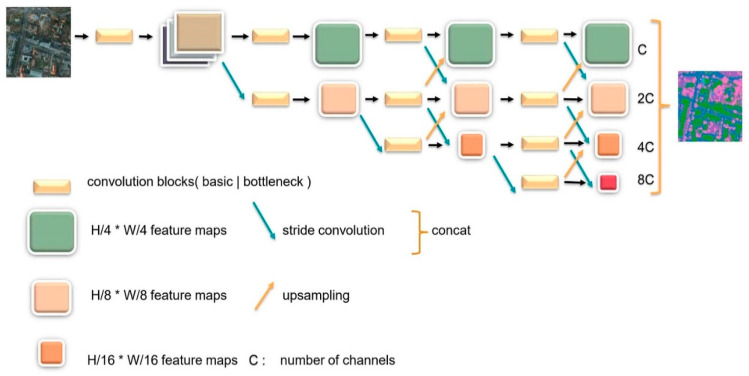
Overview of HRNet. Multi-resolution convolutions are connected in parallel with interactive fusion modules to output the final feature maps.

Many researchers have widely utilized convolution operations in series to compute low-resolution representations and then gradually recover high-resolution representations. Typical networks designed following this rule are U-Net, SegNet [47], etc. A drawback of these networks is the loss in the position sensitivity of the representation, and the decoder part can hardly recover all the information. In order to learn the high-representation and reduce the loss in spatial accuracy, HRNet [27] adopts the policy of maintaining the high resolution during the whole feature extraction procedure. The architecture is composed of several modules which are multi-resolution convolution modules in parallel, the interactive fusion module, and the representation head module. Figure 3 below depicts the whole body of HRNet.

HRNet has four stages of high to low resolution convolution. Each stage consists of group convolutions which are performed on a separate part of the input channels in order to focus on different spatial resolutions. As Figure 3 illustrates, the later stage has feature information from the previous one as well as larger receptive fields by appending extra convolution branches. Interactive fusion modules exist at the junction between each stage. Input features from the prior stage are connected with the output in a manner similar to a fully connected layer. Thus, information can be transferred between different multi-resolution representations. Through repeated fusion modules, the high-resolution branch gains strong semantic information from a low-resolution branch while the lower branch acquires highly precise spatial information from the higher one. In this way, intersections between each branch bring advantageous features mutually and eventually output stable high-resolution representation. The representation head module connects four output feature maps along the channel dimension by means of bilinear upsampling and 1 × 1 convolution operation to form a mix. HRNet fixes its depth and divides this structure into several blocks, each of which is organized by several repeatable parts, whereas the width of it is changeable for the convenient tuning capacity. 

### 3.3. Class-Oriented Region Attention Module

**Figure 4:**
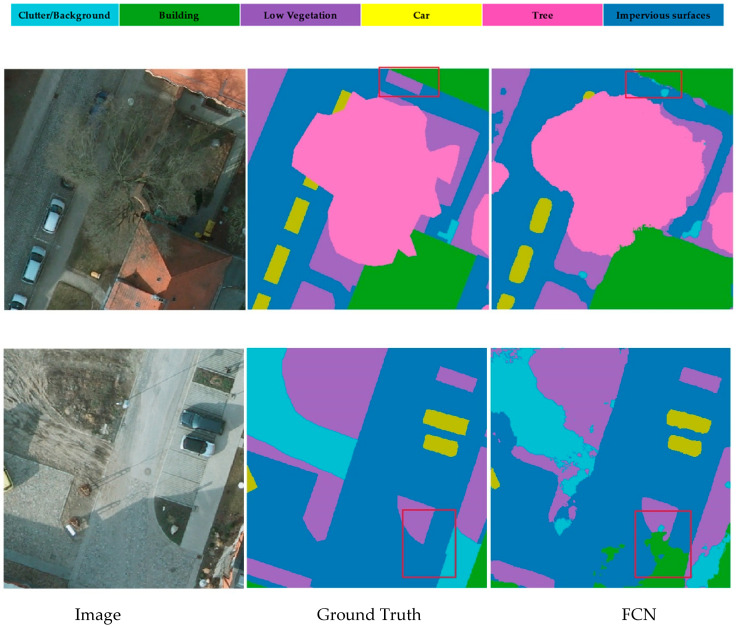
FCN model does not take the surrounding context and specific output category into consideration. Thus, there exists some mismatched relationship during inference.

The goal of semantic segmentation is to identify which category each pixel in an image belongs to; the label value of each pixel directly influences the recognition of the targeted object, and the information as well as contextual relationships around the pixel play a decisive role in the understanding of complex scenes. Since the model tends to input the final output category information in the last few layers, it often encounters mismatched relationships in the actual recognition problem due to the loss of contextual information. As shown in Figure 4 below, common sense usually indicates that buildings always have a regular outline instead of scattered dot shapes, yet in the figure below, the FCN [48] model cannot correctly identify these and replaces the low vegetation with the clutter. If the final output category information is combined with the contextual semantic regions in advance of the model classification and simultaneously outputs a class-oriented semantic segmentation feature map, it will greatly improve the final model classification. Influenced by these points of view [22,30,34,43,49], we applied the Class-Oriented Region Attention Module (CRAM) to roughly focus on output categories for the following semantic segmentation procedure.

CRAM is used for the exploration of the relationship between various patch regions and classes in an image. 

**Figure 5:**
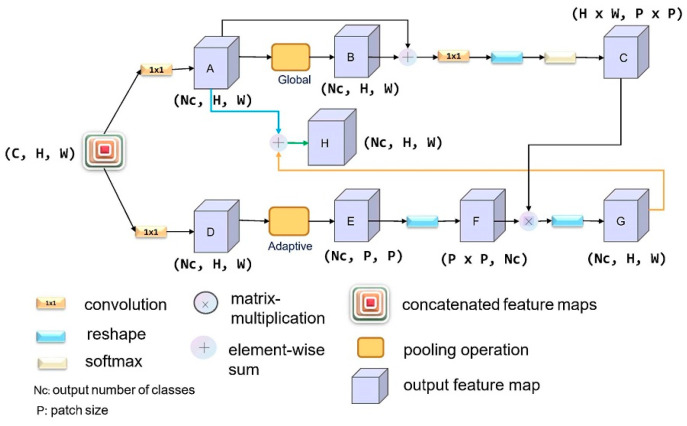
Illustration for the architecture of CRAM.

Formulation: Let us take the post-concatenated feature map $X(X\in R^{C\times H\times W})$ of the HRNET output as an example. As shown in Figure 5 above, CRAM is divided into upper and lower branches. The upper one is used to calculate the impact of pixels at different locations on the classification of object classes, while the lower one is employed to extract approximately Nc classes of objects from the original image (roughly splitting the image into several regions, $\left[O_{1},O_{2},\ldots ,O_{\mathrm{Nc}}\right]$). In this case, we can explore the relationship between different pixels and different channels over a long range from the perspective of the number of categories in the final output, enabling the model to obtain a better understanding of the global semantic information. We assume that $x_{i}$ is a regional feature in B. The purpose of using CRAM is to have an output of Nc category-based semantic segmentation feature maps while incorporating the context around the pixel. In this way, the model could pay special attention to different patch regions. The formula is defined as C, where α is the correlation coefficient between various regions in the image, an attentional map, and δ is the method of fusion transformation to output the ultimate feature map. Here, i and the number of output categories are associated with specific pixel features, and j is related to the index of positions in X.

Lower branch: We first adopt a 1×1 convolution to reduce the number of channels to Nc and then append the adaptive pooling layer to reduce the output size to P×P. Lastly, we use a series of transformation and transposition operations to obtain Nc local region $O_{j}$ of P×P size.

Upper branch: The following steps can be represented as the equation below; β is a list of operations including 1 × 1 convolution, reshaping as well as softmax, γ indicates 1×1 convolution and reshaping:

(1) $$\begin{array}{l} W_{i}=\beta \left(conv(x_{i}),\rho \left(conv\left(x_{i}\right)\right)\right)\\ \;=softmax\left(\gamma \left(x_{i}\right)\right)\\ \;=softmax\left(\gamma \left(conv(x_{i}\right)+\rho \left(conv\left(x_{i}\right)\right)\right))\\ \;\begin{array}{c} =\frac{exp\left[conv(x_{i}),\rho \left(x_{i}\right)\right]}{\sum_{i}^{N}exp\left[conv(x_{i}),\rho \left(x_{i}\right)\right]} \end{array} \end{array}$$

After a series of operations (ρ) such as 1×1 convolution, global pooling, and pixel element-wise addition, the local feature $x_{i}$ in the connected feature map X can be better grasped by the model while preserving the detailed features of the pixels to the utmost extent, and eventually dimensional matching with the extracted feature map from the lower branch can be completed by the operation of distortion.

Ultimately, we combine the results from the upper and lower branches using the softmax method by extending the fusion part:

(2) $$y_{i}=\delta \left(W_{i},O_{j}\right)+conv(x_{i})$$

From the equation above, we could obtain the output feature map $y_{i}$ with a shape of $N_{c}\times H\times W$ using the Class-Oriented Region Attention Module to focus on the relationship between patch regions and classes.

### 3.4. Class-Oriented Context Fusion Module

**Figure 6:**
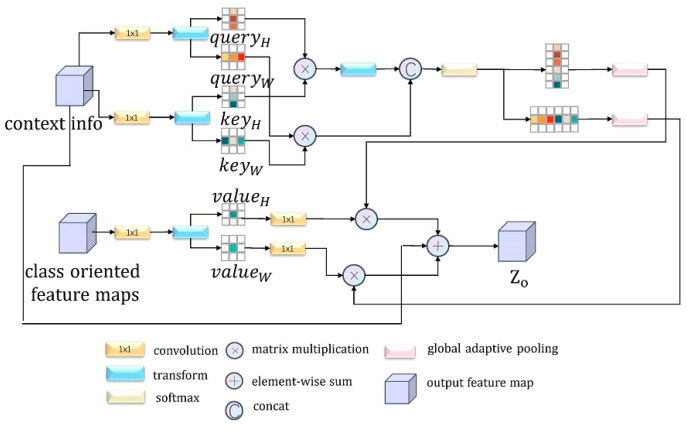
Illustration for the architecture of CCFM.

Depending on the Nonlocal [30] and OCRNet [34] structure, we developed CCFM to analyze the association between output classes in semantic segmentation and specific pixels in the image. In the previous phase, we used CRAM to explore the relationship between the output class and different area patches in the image and roughly obtained the local contextual information in the original image and the feature map aggregated in the output class. However, due to the loss of local pixel details in the process, we combined the context information with the output class-centered feature map obtained from the previous module and added a cross-attentive structure to obtain CCFM according to the research ideas in CCNet [35], which can generate sparse H+W−1 weights with a small amount of GPU memory. The attention map is a fine fusion of the two feature maps obtained in the previous phase. With multiple iterations, the model can focus on key output categories in the original image in time, while also incorporating local contextual information around all pixels, eventually exploring long-range dependencies from all positions. The design details of the module are shown in Figure 6 below.

As shown in Figure 6, it is assumed that for the obtained contextual information feature map $X_{ct}\in R^{C\times H\times W}$ and class-oriented feature maps $X_{co}\in R^{C^{\prime }\times W^{\prime }\times H^{\prime }}$, the specific computational procedure is briefly summarized as follows:

1. Apply convolution to $X_{ct},\;X_{co}$ to obtain the values of Query, Key, and Value, and then after a series of permutation and reshaping operations, obtain the value corresponding to the horizontal and vertical direction separately; these are called $query_{H},query_{W},key_{H},key_{W},{value}_{H},{value}_{W}$.
  (3) $$\left(query_{H},query_{W}\right)=\delta \left(X_{ct}\right)$$
  (4) $$\left(key_{H},key_{W}\right)=\delta^{\prime }\left(X_{ct}\right)$$
  (5) $$\left({value}_{W},{value}_{W}\right)=\delta^{\prime \prime }\left(X_{co}\right)$$
2. Perform the matrix product operation on the Query and Key in the matching direction. When doing the cross-attention operation, because there will be an overlapping element in the horizontal and vertical directions, we can generate a matrix with all diagonal elements being negative infinity. $M_{-inf}$ is summed with the product matrix of the query and key in the vertical direction, so that the subsequent softmax operation sets the repeated elements to 0, and after a series of non-local operations, the corresponding attention map is obtained. The above operation can be expressed by the following formula:
  (6) $$\left(attention_{H},attention_{W}\right)=\mathrm{softmax}\left(q_{H}^{T}\cdot k_{H}+M_{-inf},q_{w}^{T}\cdot k_{w}\right)$$
3. Match the attentional map with the values horizontally and vertically: we perform a series of transformations between the two matrixes, including the use of a global adaptive pooling layer GAP and the convolution δ of 1×1:
  (7) $$\left(attention_{H}^{\prime },attention_{W}^{\prime }\right)=GAP\left(attention_{H},attention_{H}\right)$$
  (8) $$\left(value_{H}^{\prime },value_{W}^{\prime }\right)=\delta \left(value_{H},value_{W}\right)$$
4. Merge the transformed attention map, Value, and the original contextual information. The purpose of adding original contextual information at the end is to implement a residual link-like operation, allowing the network to review the global information again:
  (9) $$Z_{o}=\omega \cdot \left[\varphi \cdot \left(attention_{H}^{\prime }\times value_{H}^{\prime }\right)+\varphi \cdot \left(attention_{W}^{\prime }\times value_{W}^{\prime }\right)\right]+X_{ct}$$
5. Given that the context information obtained by CCFM comes from both vertical and horizontal directions, the extent of the association between the main pixels and their surrounding parts, the $N_{c}$ outputs, is still limited. We thus adopt a sequential CCFM to compensate for the shortcomings of a single cross-attention mechanism, so that more compact and rich semantic information can be better harvested from all pixels:
  (10) $$Z_{o}^{\prime }=f_{CCFM}\left(Z_{o},X_{co}\right)$$

### 3.5. Mish Activation

In order to enable the neural network to deal with more complex feature information, researchers need to add activation functions to the model to increase its nonlinear characteristics. 

The unilateral suppression of the Relu function makes the neurons in the model sparsely activated, so it is often used to improve the performance of neural networks. Here, we chose the Mish [50] activation function due to its own series of properties, e.g., the upper limit is unbounded and the lower limit is bounded, smooth, and non-monotonous—when applied to the model, it can provide a boost in performance. The formula of the Mish activation function is as follows:

(11) $$f\left(x\right)=x\cdot \tanh\left(\ln\left(1+e^{x}\right)\right)$$

**Figure 7:**
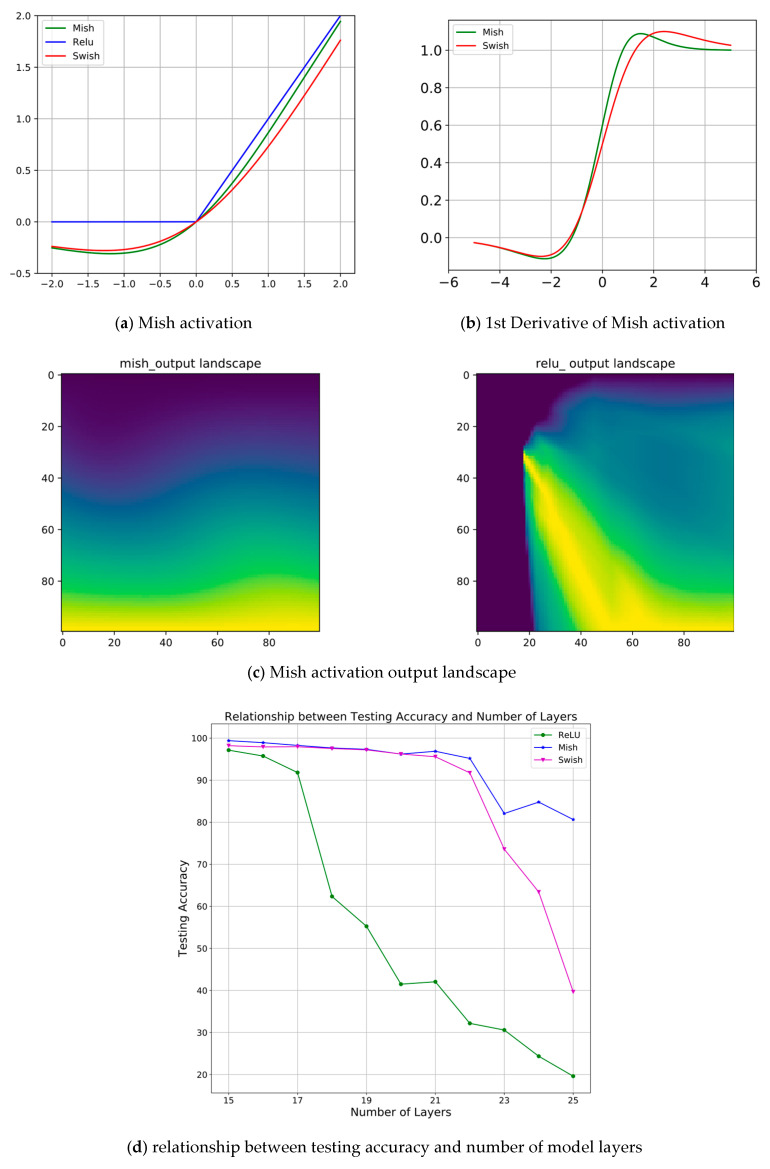
Graph to show the attributes of Mish activation.

The following is based on Figure 7:

(a) The upper limit is unbounded (positive values can reach any height), which can prevent model saturation.
(b) A small amount of negative values can make the model have a better gradient flow, so as to avoid the hard zero bound of Relu.
(c) Is the output landscape of a model using Mish and Relu activations. It clearly reveals the sharp transition of the data magnitude in the Relu model contrasting to mish.
(d) The smoother property makes the data information better transferred to the deeper layers of the neural network to ensure the generalization and accuracy of the model.

## 4. Experiments and Results

### 4.1. Datasets

#### 4.1.1. Overview

**Figure 8:**
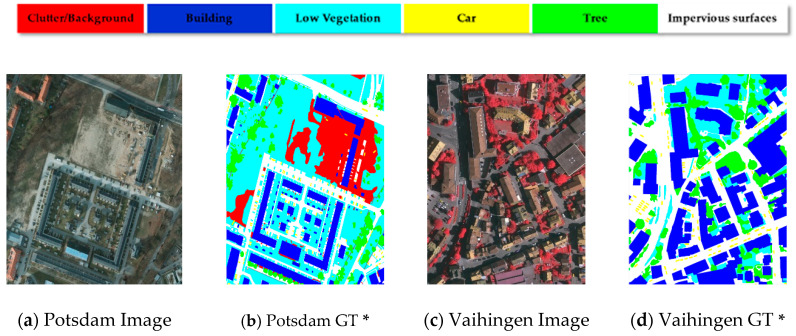
Images and Ground Truth of Potsdam and Vaihingen Datasets (* GT: ground truth).

In order to verify the effectiveness of the proposed method, we verify the model on two high-quality datasets, namely, the Potsdam and Vaihingen datasets. Figure 8 shows an overview of the dataset. To make a reasonable comparison of the differences and advantages of different models using the benchmark dataset, ISPRS proposed the two datasets in the 2d semantic labeling competition, which enables researchers to promote the development of semantic segmentation and remote sensing. The sampled images of the two datasets cover slightly different areas. The Potsdam dataset is oriented around complex urban situations, while Vaihingen covers small villages.

##### Potsdam

Potsdam consists of 38 high-resolution images with a resolution of 6000 × 6000 pixels and a ground sampling distance (GSD) of 5 cm. A total of 24 images are used for model training, and the other 14 are used to verify different model algorithms. The dataset supplies sampled images consisting of three different channels, including IRRG (IR-R-G), RGB (R-G-B), RGBIR (R-G-B-IR), as well as DSM and NDSM (Normalized digital surface models) files. There are six categories, namely, impervious roads, buildings, low-density shrubs, trees, cars, and scattered background information, including some rivers and water and some objects that appear to be of great contrast as well as low importance to other types (for example, containers, tennis courts, swimming pools). 

##### Vaihingen

The sampling area in Vaihingen is a small village with 33 images, 16 of which are used for model training and 17 for model testing. The image resolution fluctuates around 2500 × 2000 pixels, the GSD value is 9 cm, and the flight height at the time of sampling was 900 m. The dataset provides three-band IRRG (IR-R-G) images, which are similar to the Potsdam dataset, including six categories with five foreground objects and one background object.

#### 4.1.2. Data Preprocessing

**Figure 9:**
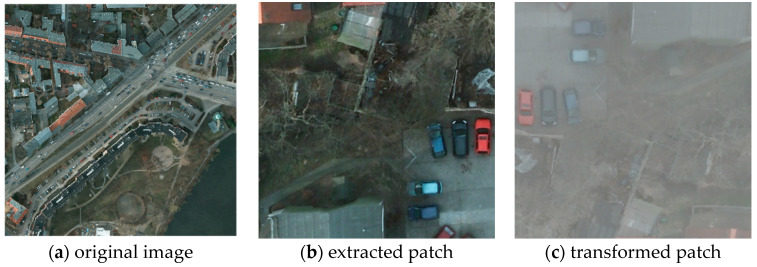
(**a**) is the original image with 6000 × 6000 pixels. (**b**) is the extracted patch with 512 × 512 pixels from the original input image and (**c**) is the transformed one with flip, brightness, and contrast change operations, etc.

Among the 24 high-definition images with label information used for model training in the Potsdam dataset, we only used 23 images excluding one image whose id is “top_potsdam_4_12” and divided the remaining images into a training set and validation set consisting of 18 and five photos, respectively. The remaining 15 images in the Potsdam dataset were used for model testing. Similarly, the 16 annotated images in the Vaihingen dataset were randomly split into the training set and the validation set composed of 13 and three images, respectively. Because the number of samples in the training set and the test set was too small, we needed to augment the original data set. The GPU used for model training is the NVIDIA Titan XP. Due to the limitation of GPU memory (12G), we needed to extract small patches with 512 × 512 pixels from the original 6000 × 6000 pixel high-definition picture. Finally, model inference was performed on the local small image; then, the results of the inference were rebuilt into the original high-definition 6000 × 6000 pixel large image, and the same operation was performed on the training set. At the same time, we flipped, rotated, distorted, random padded, adjusted the brightness, contrast, and saturation of the image, and randomly erased local areas of the image in the training set and the validation set, so that the dataset was increased from the original about 40 times, decreasing the phenomenon of overfitting in the training process of the model. The reason why we choose to expand the original data set 40 times is that the original data set was small and easy to cause over-fitting of the model; 40× amplification not only ensured the accuracy but also took efficiency of training into account. There were 38 images in the Potsdam dataset, of which 24 had annotations, excluding one photo with a wrong label, and we used 23 of them for model training and the remaining 14 unlabeled images for model testing. Vaihingen had 33 images with 16 annotated patches. The remaining 17 unlabeled images were used for model testing. The two datasets are processed separately and did not affect each other. Figure 9 shows the original image and the extracted patch along with the corresponding augmented one.

### 4.2. Evaluation Metrics

To evaluate the performance of the proposed model, we used the F1 score and Overall Accuracy (OA) for classes excluding the background in the image. The formula of F1 score and OA is defined as follows:

(12) $$Precision=\frac{TP}{TP+FP},\;Recall=\frac{TP}{TP+FN}$$

(13) $$F1=2*\frac{Precision*Recall}{Precision+Recall}$$

(14) $$\mathrm{OA}=\frac{TP+TN}{TP+FP+TN+FN}$$

During the equations above, *TP*, *TN*, *FP*, and *FN* mean true positive, true negative, false positive, and false negative, respectively. 

### 4.3. Implementation Details

**Figure 10:**
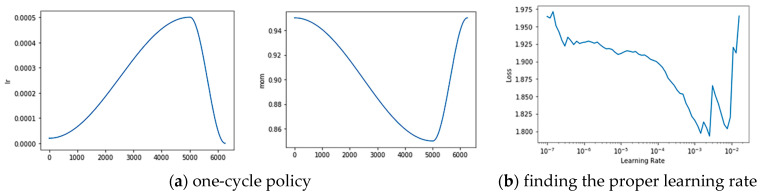
Using the one-cycle policy to find the proper learning rate.

We trained the model on two Nvidia Titan XP GPUs (12 GB memory and 64 GB RAM) with mixed precision, synchronous batch-normalization, and data parallel distribution tricks. All the proposed method and models were implemented using pytorch and fastai [51]. Due to the limitation of the memory size and high-resolution image, we cropped the input data with a small sliding window with 512 × 512 pixels before the model training process. During the stage of model inference, we made a prediction on an image with 512 × 512 pixels and rebuilt the 6000 × 6000 image using the extracted patches for the evaluation. The models use the initializing weight pretrained on the ImageNet dataset. We adopted the one-cycle policy put forward by Leslie N. Smith [52] in order to find the appropriate learning rate. As the curve and policy suggested, we set the initializing learning rate, weight decay, and momentum parameter to 0.004, 2 × 10^−4^, and 0.9, respectively, with batch size five per GPU. Cross-entropy loss was employed to analyze the difference between the ground truth and inference results. Models were trained for 200 epochs for convergence. To verify the robustness of our model and to compare it with other state-of-the-art methods, we added k-fold cross-validation based on the Vaihingen dataset. Considering the size of the dataset and the accuracy and efficiency of the model training, we set the k-parameter as the commonly used number 10 in the machine learning domain. Specifically, we partitioned the 16 images with annotation information in Vaihingen into ten consecutive copies, each of which was treated as a test set once, and the rest was taken as the training set of the model. Finally, we calculated the final recognition overall accuracy by obtaining the average of the ten results. Figure 10 shows the details of several tricks used during training.

### 4.4. Results

#### 4.4.1. Visualization Results

**Figure 11:**
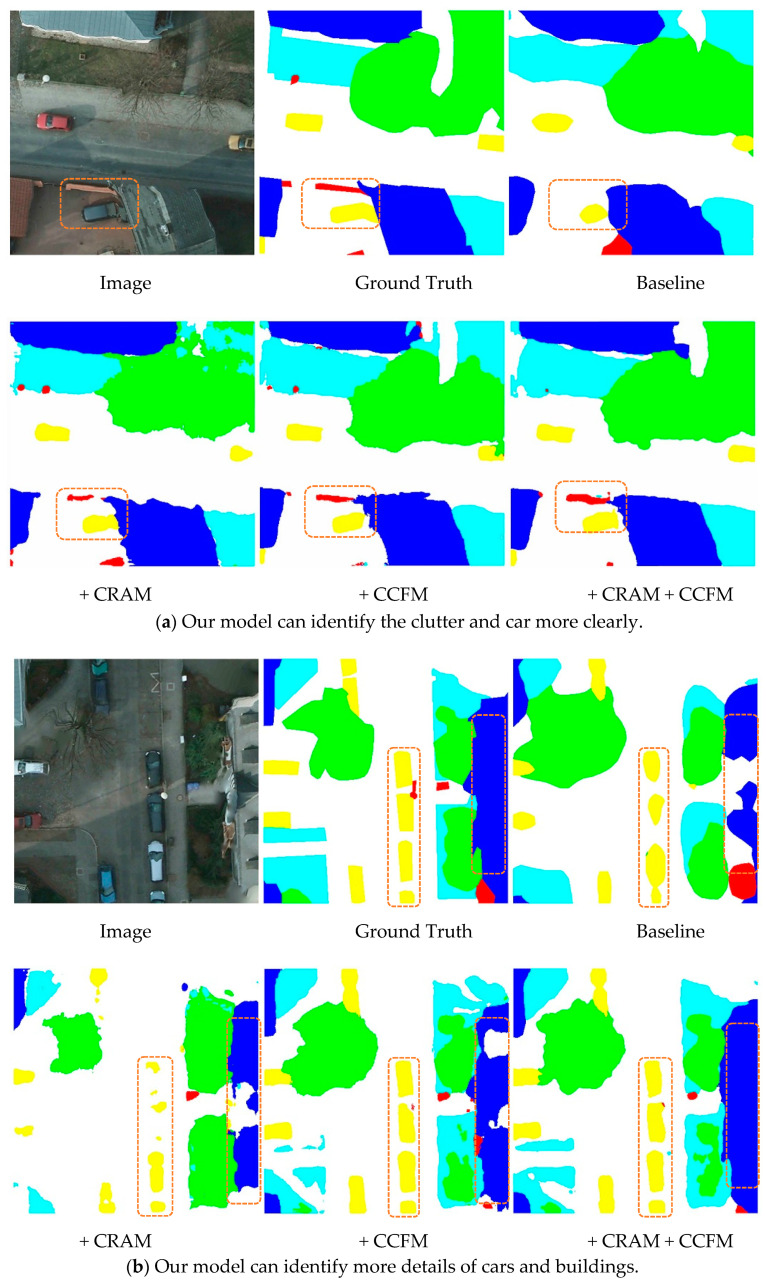
Visualization of the classification map generated by different modules.

To illustrate the effectiveness of the model, we selected two sets of images from the Potsdam dataset for qualitative display. Each set contained a 512 × 512 representative sample of the original image, the real labeling information, and the semantic segmentation map of the different model outputs. The baseline model was HRNet, and based on that, we included CRAM and CCFM. The two modules were finally integrated into the baseline simultaneously to demonstrate the effect. The position of the orange solid line box in the figure below represents an area that could not be well identified in the basic model. We can clearly observe that in Figure 11a, because the clutter background area in the box was smaller than the surroundings, it was difficult to find the clutter in the baseline, but after adding CRAM and CCFM, the model gradually uncovered the clutter area and adjacent target so that they could be properly labeled. In Figure 11b, the positions in the two vertical boxes were more or less missing in the semantic segmentation map output from the backbone, while the model with integrated modules better identified the outer contours of buildings and cars in detail due to a better understanding of the semantic relationships between different areas and pixels, thus avoiding the common problem of mismatching relationships. Therefore, our approach has a great advantage over the basic backbone, as it can recognize the details of the outer contour of an object and at the same time focus better on the global relationship between different regions and pixels.

#### 4.4.2. Ablation Study

**Figure 12:**
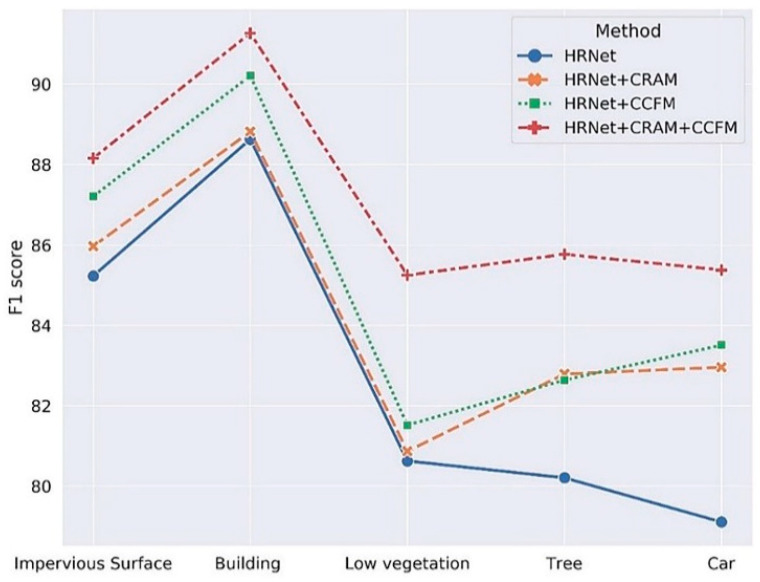
Visualization of ablation study results of CRAM and CCFM.

To quantitatively verify the validity of our model, we performed ablation tests on the Potsdam validation set. In the experiment, we used the HRNetV2-W48 model pretrained on ImageNet as the backbone [27] and added each sub-module to verify functionality individually. When using HRNet + CRAM, we directly took the output feature map of CRAM and the output value of the 1 × 1 convolutional layer as the final output; when using the HRNet + CCFM alone, we substituted part of CRAM with a 3 × 3 convolutional layer, and the number of channels remained identical. As CRAM can enhance the connection between different sub-regions and the final output category, and CCFM enables the model to concentrate more on the specific pixels in the image as well as the global information in the final output, after incorporating CRAM and CCFM, it was evident that the F1 score of each output category improved (*: Imp Surf means the impervious surface, Low veg means low vegetation). The significant performance improvement was the car recognition in the original image. As the target car was tiny, detailed semantic information is prone to be lost in the process of semantic segmentation. The addition of CRAM provided an excellent way of capturing information on objects of various sizes, while using CCFM to extract global information in a long range context, thus boosting the F1 score for car identification from 79.11% to 85.38%. Table 1 shows ablation study result in the Potsdam Dataset of different modules. Figure 12 visualizes the ablation study result.

**Figure 13:**
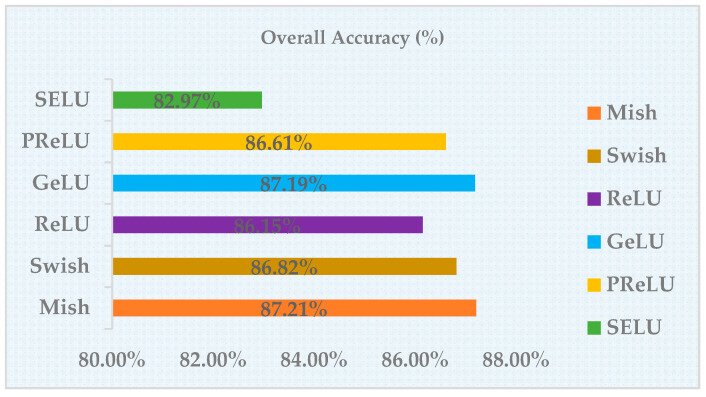
Visualization of ablation study result of activation functions.

We also performed the ablation study on the activation functions. The selected comparison objects were mainly the following activation functions: Mish, Swish, SELU, ReLU, PReLU, GeLU. To make the results more reliable, we only replaced the activation function in the network with Mish, and the other hyperparameters were kept constant; the results of the experiment on the Potsdam dataset are shown in the following table. It can be concluded that the robustness of the Mish activation function was better, which can improve the model performance for semantic segmentation in remote sensing images. Table 2 shows ablation study result of different activation functions. Figure 13 visualizes the ablation study result.

## 5. Discussion

**Figure 14:**
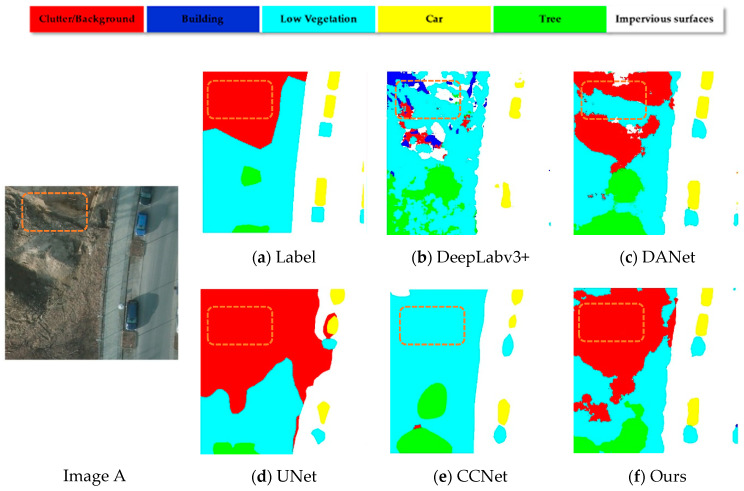

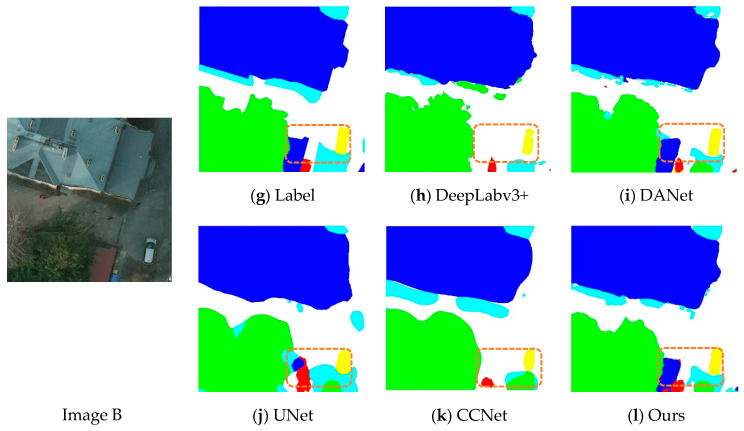
Qualitative comparisons between different methods applied to sample images.

In order to verify the validity and precision of our method, we selected several state-of-the-art methods in the field of semantic segmentation and evaluated the pros and cons of different methods by conducting experiments, specifically adopting the following approaches: FCN, UNet [18], UNet [18] + SEA [31], DeepLabV3+ [20], CCNet [35], and DANet [33] and qualitatively show the performance of the model in Figure 14. From Figure 14, it can be concluded that DANet, CCNet, and our methods are significantly better than the other methods chosen because all three employ an attention mechanism that allows the model to better incorporate local detail features in the image with global semantic information. From Figure 14c,e,f below, we can observe that the part of the box region in orange looks like a low bush, and both DANET and CCNET misidentified this area, while our method correctly identified it. From Figure 14h,j,l, it can be noted that CCFM’s attention to the relationship between the output category and the global pixels enabled our model to have a greater advantage in recognizing the detailed outer contours of objects, while the DeepLabV3+ and UNet models in Figure 14 could not preserve the spatial location information well in the input image and failed to restore all the information of the original in the upsampling process. Neither had a good recognition of the objects in the frame. 

**Figure 15:**
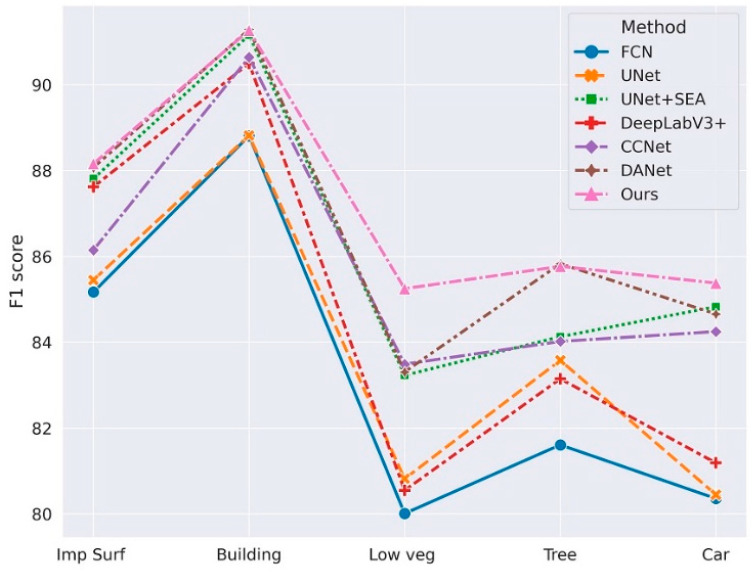
Visualization of comparison study results for Potsdam.

We present a quantitative analysis in the following Table 3. From Table 3, it can be noticed that several models considering attention mechanisms (UNet + SEA, CCNet, DANet in Table 3) exhibited greater superiority in the recognition of small target objects (cars, etc.) in remote sensing images, where the UNet + SEA model had a 4.37% improvement in the F1-score compared to UNet alone for semantic segmentation. The performance of CCNet and DANet was comparable, while our model improved the final average F1-score and OA over the other approaches by taking into account the effects of the relationship between output categories and segmented local patches as well as the global semantic information. Although our model had a great margin in the segmentation of cars, it had minor advantages compared with the others in the recognition of buildings. This may be attributed to the covered trees on the roof which lowered the performance. Table 3 shows comparison study result of different state-of-the-art methods. Figure 15 visualizes the comparison study result.

**Figure 16:**
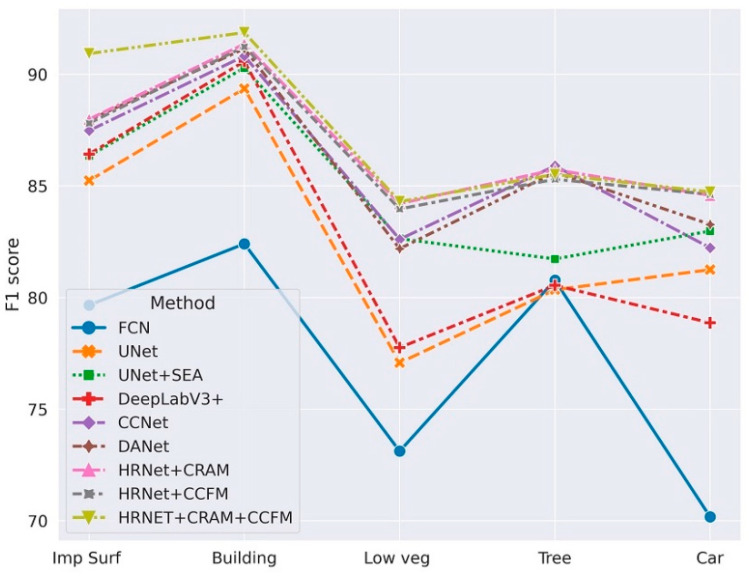
Visualization of comparison study results for Vaihingen.

We also conduct a comparative analysis of the state-of-art models on the Vaihingen dataset. To better illustrate the robustness of the models, we use k-fold cross-validation as mentioned in Section 4.3. We set k = 10 and divided the labeled training data into ten consecutive folds. The specific results are shown in the following table, from which we can conclude that the backbone, after combining CRAM and CCFM, significantly improved the recognition ability of small target objects, and the recognition accuracy of subcategories of low vegetation, impervious surface, and car was enhanced more obviously, while the overall accuracy also improved. This indicates that the model can perform a better analysis of the association between objects by integrating the semantic context information with different locations in the image, thus accurately recognizing objects in the remote sensing image. Table 4 shows comparison study result of different state-of-the-art methods on Vaihingen Dataset. Figure 16 visualizes the comparison study result of Vaihingen.

## 6. Conclusions

In this work, to address the class mismatch problem and object edge blurring that arise in the semantic segmentation problem of remote sensing images, we propose an end-to-end CNN architecture for the semantic segmentation in aerial images on the backbone of HRNet integrated with CRAM and CCFM to present an end-to-end semantic segmentation of remote sensing images. HRNet reduces the loss of spatial accuracy by means of parallel and interactive fusion branches. HRNET is able to fuse multi-scale features using parallel interactive convolutional branches to reduce the loss of spatial location information. CRAM first partitions several sub-regions and acquires the obtained local and global representations to estimate the relationship between the different classes and each sub-region after a series of pooling and other operations. Using the strategy of nonlocal and cross-like attention, CCFM can access the long-range dependency and perform weighted summation for the feature information at the outer and distal positions, thus well exploring the relationship between the class and specific pixel in the image. We lastly performed an ablation study and k-fold validation on the ISPRS 2D Semantic Labeling Contest dataset. This verified the robustness of our method and demonstrated the feasibility and accuracy of our method compared with the state-of-the-art methods.

## Figures and Tables

**Table 1:** Ablation Study Result of different modules in the Potsdam Dataset.

Method	Per-Class F1 Score (%)					mean F1 (%)	OA (%)
	Imp Surf *	Building	Low Veg *	Tree	Car		
HRNet	85.23	88.63	80.63	80.21	79.11	82.762	87.21
HRNet + CRAM	85.97	88.82	80.87	82.79	82.96	84.282	87.86
HRNet + CCFM	87.21	90.21	81.52	82.64	83.51	85.018	88.32
HRNet + CRAM + CCFM	88.16	91.27	85.25	85.77	85.38	87.166	89.95

(*: Imp Surf means the impervious surface, Low veg means low vegetation).

**Table 2:** Ablation Study Result of different activation functions.

Activation Function	Epoch	Learning-Rate	Batch-Size	Optimizer	Backbone	OA (%)
Mish	200	0.004	5	Adam	HRNet	87.21%
PReLU	200	0.004	5	Adam	HRNet	86.61%
GeLU	200	0.004	5	Adam	HRNet	87.19%
ReLU	200	0.004	5	Adam	HRNet	86.15%
Swish	200	0.004	5	Adam	HRNet	86.82%
SELU	200	0.004	5	Adam	HRNet	82.97%

**Table 3:** Comparison with the state-of-the art on the Potsdam Dataset.

Method	Per-Class F1 Score (%)					Mean F1 (%)	OA (%)
	Imp Surf *	Building	Low Veg *	Tree	Car		
FCN	85.17	88.81	80.01	81.61	80.36	83.192	86.04
UNet	85.45	88.82	80.82	83.58	80.45	83.824	87.82
UNet + SEA ^※^ [31]	87.81	91.16	83.23	84.13	84.83	86.232	89.32
DeepLabV3+ [20]	87.62	90.50	80.55	83.15	81.19	84.602	88.74
CCNet [35]	86.15	90.64	83.49	84.02	84.25	85.71	89.28
DANet [33]	88.07	91.29	83.31	85.83	84.66	86.632	89. 87
Ours	88.16	91.27	85.25	85.77	85.38	87.166	89.95

(^※^: SEA is the Self-Attention method used in [31]. *: Imp Surf means the impervious surface; Low veg means low vegetation).

**Table 4:** Comparison with the state-of-the art on the Vaihingen Dataset

Method	Per-Class F1 Score (%)					Mean F1 (%)	OA
	Imp Surf *	Building	Low Veg *	Tree	Car		
FCN	79.66	82.41	73.12	80.78	70.18	77.23	79.32
UNet	85.24	89.36	77.08	80.35	81.25	82.656	81.55
UNet+SEA ^※^	86.35	90.29	82.64	81.73	82.98	84.798	88.97
DeepLabV3+	86.43	90.58	77.76	80.55	78.87	82.838	85.26
CCNet	87.48	90.82	82.61	85.9	82.23	85.808	86.61
DANet	87.95	91.15	82.19	85.61	83.28	86.036	87.21
HRNet + CRAM	87.99	91.36	84.21	85.73	84.58	86.774	88.93
HRNet + CCFM	87.81	91.24	83.97	85.3	84.62	86.588	89.02
HRNET+CRAM+CCFM	90.93	91.87	84.3	85.52	84.73	87.47	89.17

(^※^: SEA is the Self-Attention method used in [31]. *: Imp Surf means the impervious surface; Low veg means low vegetation).

## Nomenclature

CNN	Convolutional Neural Networks
FCN	Fully Convolutional Networks
GSD	Ground Sampling Distance
CRAM	Class-Oriented Region Attention Module
CCFM	Class-Oriented Context Fusion Module
F1	F1 score
OA	Overall Accuracy
